# Development and Implementation of a Birth Forecasting Tool to Optimize Resources in Obstetrical Care During the COVID-19 Pandemic: Mixed-Methods Study

**DOI:** 10.2196/68284

**Published:** 2025-08-22

**Authors:** Huibert-Jan Joosse, Karin Jongsma, Marcel Moes, Kitty W Bloemenkamp, Wouter M Tiel Groenestege, Wouter W van Solinge, Saskia Haitjema, Maarten B Kok

**Affiliations:** 1 Central Diagnostic Laboratory, University Medical Center Utrecht, University Utrecht, Heidelberglaan 100, Utrecht, 3584CX, The Netherlands, 31 88 755 0759; 2 Department of Bioethics & Health Humanities, University Medical Center Utrecht, Utrecht University, Utrecht, The Netherlands; 3 Saltro Diagnostic Center, Unilabs Netherlands, Utrecht, The Netherlands; 4 Department of Obstetrics, Division Woman and Baby, Wilhelmina Children's Hospital, University Medical Center Utrecht, Utrecht, The Netherlands; 5 Network Regional Acute Obstetric Care (Regionaal Acuut Zorg Overleg Midden Nederland), Utrecht, The Netherlands; 6 Digital Innovation Team, Unilabs Group, Geneva, The Netherlands

**Keywords:** resource allocation, regional health planning, quality of care, routinely collected data, stakeholder participation

## Abstract

**Background:**

Medical resource allocation is important to ensure availability of care, especially in challenging circumstances like a pandemic. In fields of unpredictable care demand such as obstetrics, forecasting may help manage scarce resources.

**Objective:**

The development, validation, and implementation of a regional birth forecasting tool to support obstetrical staff planning in the Utrecht region during the COVID-19 pandemic.

**Methods:**

We combined predicted birth dates retrieved from Saltro, a large regional primary care laboratory, with data from the Dutch national perinatal registry (Perined) and Statistics Netherlands for model development. We created and implemented an HTML tool visualizing these forecasts, which were discussed during the regional acute obstetric health care network meetings. Six months after implementation, we assessed the impact of the tool using an evaluative stakeholder meeting.

**Results:**

We achieved a performance accuracy (*R*) of 0.45, 0.61, and 0.67 (all actual number of births within 95% CI) forecasting the number of births in the region, pooled in 1-, 2-, and 3-weekly bins, respectively. After presenting these findings to stakeholders, we implemented a forecasting tool using the 2-week bin model. The evaluative stakeholder meeting proved that the tool improved communication, awareness of health care need, and collaborations among health care providers in the Utrecht region. Additionally, stakeholders identified additional applications, such as communication with patients and training of obstetric health care providers.

**Conclusions:**

Implementation of a forecasting tool for the number of births based on available data across the health care system added value to obstetrical care by providing insight into care demand, and increasing communication, awareness, and collaboration between health care providers. Further research should aim at improving regional obstetric acute care by fostering data sharing in order to improve health care demand forecasts.

## Introduction

Clinical care for patients in hospitals, and especially in the acute care chain, depends on the infrastructure and on careful planning of resources such as staff, equipment, and beds. The COVID-19 pandemic magnified this complexity as the combination of patients who were hospitalized with COVID-19 and frequent sick leave of already scarce medical personnel for clinical care posed a burden on hospital capacity. Even more complex is planning in acute care departments, including the emergency departments, the obstetrics ward, and the intensive care where medical personnel are not only scarce, but patients’ needs are unpredictable [1,2]. The efficient allocation of resources is an important performance indicator of quality of care (QoC) according to the US Institute of Medicine [3]. The World Health Organization (WHO) identified the impairment of QoC in obstetrics as a major roadblock for ending preventable maternal and neonatal mortality and morbidity and called for skilled care and evidence-based practices [4]. Managing resources such that high-quality obstetrical care delivery can be maintained is thus essential when hospital capacity is under pressure.

Contrary to acute care needs for emergency departments, the expected date of birth provides an opportunity to predict future average demand in obstetrics. Date of birth itself can be understood as a result of a prediction model, using first day of last menstruation date, and is in the Netherlands confirmed by the crown-rump length (CRL) at ultrasound around 11 weeks of pregnancy [5,6]. In addition, the Dutch health care system offers routine first-line obstetrical care around the eighth week of pregnancy. Around 11 weeks, women are invited to participate in a screening (booking blood) for infectious diseases (HIV, Hepatitis B, and Lues), anemia, and irregular erythrocyte antibodies (eg, Rhesus disease). A total of 99% of women take part in this screening program [7]. On the lab test order form, the calculated date of birth is listed. As such, these laboratories have access to data of expected birth dates. In the midst of regional (larger Utrecht area) capacity problems in acute obstetric care, there was a need within the network regional consultation acute care (ROAZ midden Nederland) to forecast the number of births in the region. Since we were not able to extract the estimated date of delivery from the existing perinatal health care systems, the idea came up to use the data registered during the booking bloods.

The goal of this study was to support obstetrical staff planning during the COVID-19 pandemic by using data from Saltro, a large primary care laboratory in the Utrecht region of the Netherlands and regional obstetrics outcomes to develop, validate, and implement a forecasting tool for the trend in number of births. Additionally, a qualitative stakeholder meeting was held 6 months after implementation in order to discuss the impact of the tool.

## Methods

### Data Sources

In order to model (trends in) the number of births in the population, we retrieved all historical data of expected birth dates from 2014 to 2019 of Saltro, as registered during the screening around 11th week of pregnancy, and linked them to a dataset of Statistics Netherlands with live births from 2015‐ to 2017 as outcomes. To ensure a representative dataset, while also ensuring that data loss was minimized, we included only data from municipalities where Saltro had data on at least 55% of pregnancies. We aggregated the data to the number of births per week. We accounted for the uncertainty of the expected date of birth by using the national pregnancy length distribution (in weeks) of the Dutch national perinatal registry, Perined [8]. According to the Dutch legislation, this research did not fall under the Medical Research Involving Human Subjects Act (WMO). Only anonymized aggregated data were used for analysis. The research was conducted according to the declaration of Helsinki. Health care professionals provided verbal informed consent prior to the interviews.

### Forecasting Tool and Model Development

**Figure 1.**
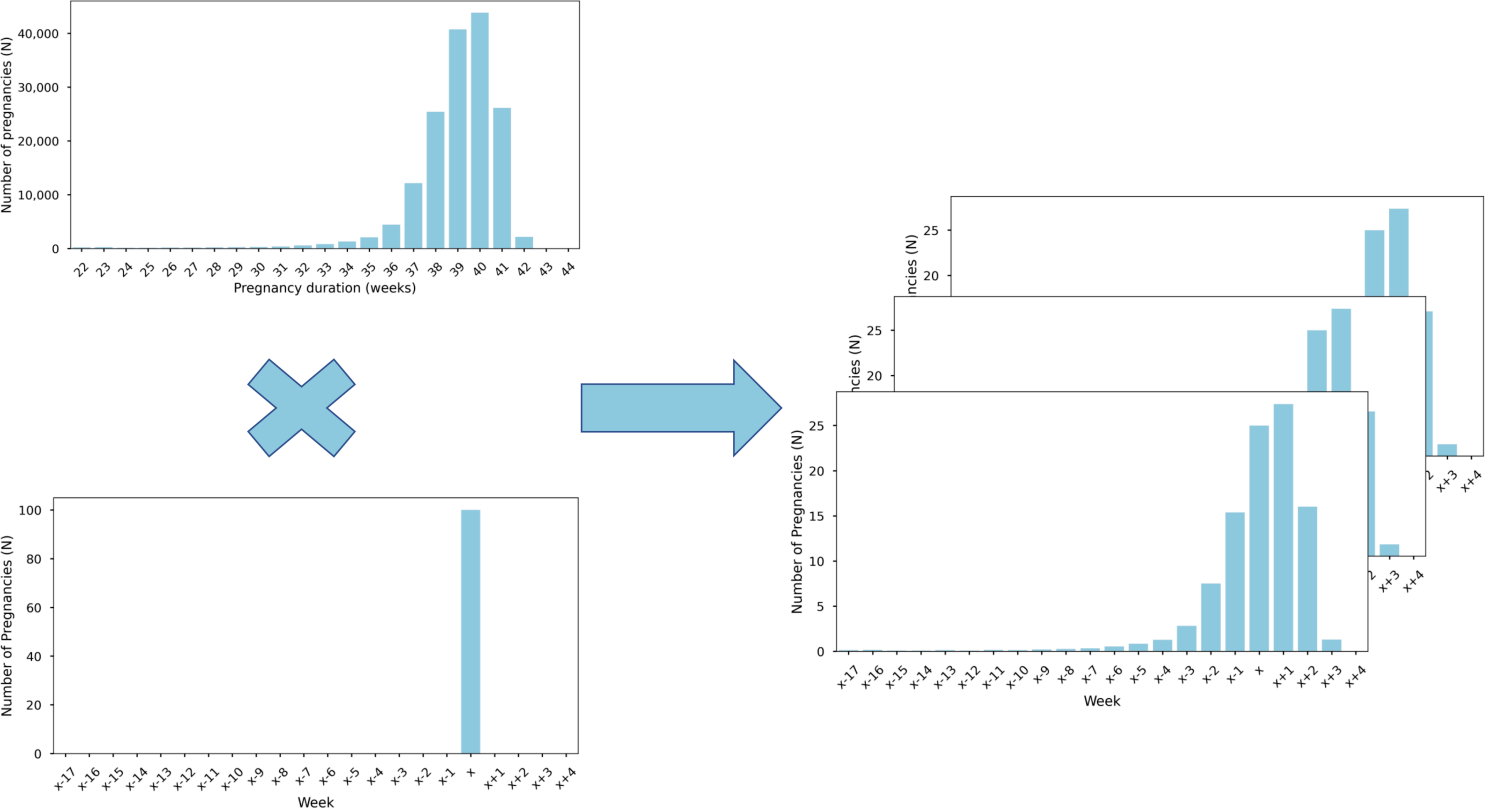
Modeling approach. For a specific week (**X**), each expected birth date (left-bottom) was assigned to a specific week using the Perined distribution of pregnancy durations (left-top). By repeating this simulation 1000 times, we were able to calculate a mean estimate and SD assigned to each surrounding week (x-17 to x+4) (right). By repeating this for all weeks in our data, we were able to estimate a cumulative estimate per week.

To correctly forecast the number of births per week, we used the expected birth date as the base model. Consequently, we distributed the number of births for each week over the surrounding weeks according to the Perined distribution (Figure 1) and repeated this process 1000 times per week. This resulted in a calculated mean and SD per week. For a certain week, the number of expected date of births would actually be forecasted in the period between 17 weeks before and 5 weeks after the denoted week. When repeating this process for all weeks, the number in every week would become a sum of all these calculations.

To validate the modeling output on actual outcome data, we correlated the mean forecasted number of births for each week with the amount as reported by Statistics Netherlands. We combined the number per week to 2- and 3-weekly bins (ie, summing up the numbers for week 1 and 2, week 3 and 4, etc), and assessed whether this improved the correlation and thus predictive accuracy. Considering the potentially high variance of calculated number of births, binning the project number of births to 2- or 3-weekly bins may help smooth out large weekly differences. To compare the mean estimate and a 95% CI of our model to the actual number of births, we normalized the visualization of these two types of data, so that these two were comparable.

This work, and the development of the resulting tool, was performed using the Python programming language (version 3.8, Python Software Foundation) and the *pandas* (Version 1.1), *numpy* (version 1.19), and *scipy* (version 1.5) packages. We used the *plotly* package (version 4.14) for interactive visualization purposes.

### Implementation Research Using Stakeholder Meetings

After the model for the tool was validated, we presented our findings to stakeholders in the obstetrics field, including future end-users of the tool, such as managers and obstetric care providers (n=13) during the weekly consultation of the regional obstetric acute care (ROAZ midden Nederland). After an introduction explaining the modeling and the visualization, we sent the forecast to this status meeting on a 2-week basis.

In order to assess the added value of the forecasting tool in the obstetrics care, we invited regional stakeholders (eg, gynecologists, midwives, and managers) from the network regional consultation acute obstetric care (ROAZ Midden Nederland) for an evaluative stakeholder meeting 6 months after implementation of the tool. To this purpose, we created a topic list to investigate the experiences with staff planning and resource management after implementation of the tool as compared to before this implementation, and the experience with use of the forecasting tool (Table S1 in Multimedia Appendix 1). We then analyzed this discussion using Nvivo (version 12.6.1.970; QSR International), and extracted information about the aforementioned topics, as well as remarks on the tool, including possible areas for further research and improvements of the tool.

### Ethical Considerations

According to the Dutch legislation [9], this research did not fall under the Medical Research Involving Human Subjects Act (WMO). Only anonymized aggregated data were used for analysis. The research was conducted according to the Declaration of Helsinki. Health care professionals provided verbal informed consent prior to the interviews.

## Results

### Tool and Model Development

In total, Saltro screening data on 28,361 pregnancies were used from 23,778 patients with births per year ranging from 5052 in 2014 to 6047 in 2017 (mean 5681 per year). The Statistics Netherlands data from the included municipalities encompassed 35,394 births with births per year ranging from 8735 in 2016 to 9051 in 2014 (mean 8849 per year). The municipalities that were included in the development of the forecasting tool are given in Table 1, along with the number of pregnancies as reported by Saltro, and the actual number of births as reported by Statistics Netherlands.

**Figure 2.**
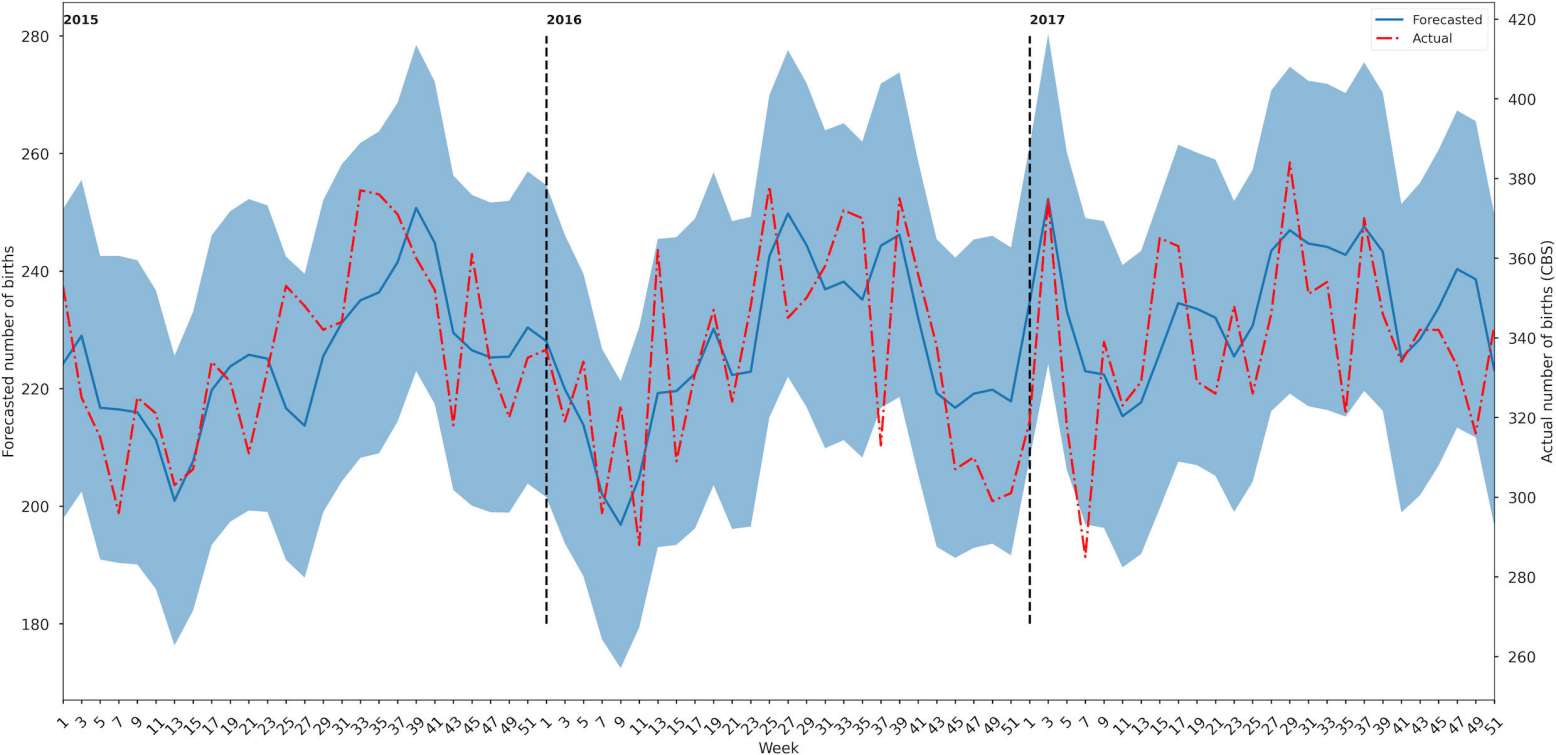
Forecast in 2-weekly bins in 2015, 2016, and 2017. The y-axis of the actual births (right-axis) is normalized on the forecast (left-axis), in order to compare the two data. The forecasted number of births is depicted in blue; the actual number of births is depicted in red.

The correlation between the modeling output and number of births as reported by Statistics Netherlands was 0.45 for weekly data. For the 2-weekly bins, we found that the correlation between the model output and the Statistics Netherlands increased to 0.61, and for 3-weekly bins, this was 0.67 (Figure 2; Figures S1 and S2 in Multimedia Appendix 2). All actual number of births were within the forecasted 95% CI, after normalization for differences in scale between modeling output and actual numbers (Figure 2).

**Table 1.** Included municipalities and included pregnancies from Saltro and reported births from Statistics Netherlands. Municipalities were included if more than 55% of reported births were found in the Saltro database.

Municipality	Expected date of births (Saltro), n	Date of births (Statistics Netherlands), n	Total births (Saltro data), (%)^a^
Bunnik	367	536	68
Bunschoten	819	1051	78
De Bilt	1148	1369	84
Houten	2011	1993	101
IJsselstein	991	1317	75
Leusden	828	1062	78
Nijkerk	1422	1784	80
Renswoude	231	305	76
Scherpenzeel	505	460	110
Stichtse Vecht	2460	2473	99
Utrecht	13,461	18,420	73
Utrechtse Heuvelrug	1239	1503	82
Woudenberg	566	590	96
Zeist	2313	2531	91

a The percentage can be more than 100%, as women can deliver in hospitals, therefore changing the municipality of birth in comparison to the municipality of the mother.

### Presentation to Stakeholders and Implementation

The stakeholders chose a 2-weekly bin for forecast-width as this was considered a good trade-off between actionability with regards to planning (smaller bins mean higher granularity), but also having good predictive power. Additionally, the numbers were presented with an overall average over the preceding years (2015‐2020). From July 2021 onwards, we provided these forecasts every 4 weeks; in September, this interval was shortened to 2 weeks upon request. We performed forecasts over 13 weeks in advance, as we observed that forecasts using this time window were most stable in comparison with follow-up forecasts, because of incomplete data in the later weeks.

### Evaluative Stakeholder Meeting

In total, 4 stakeholders attended the evaluative stakeholder meeting, including 3 stakeholders that worked in hospitals and 1 stakeholder that worked as community midwife.

#### Insights Before Introduction of the Tool

The stakeholders noted that before the introduction of the tool, there was a sense of competitiveness between different health care providers, as there was little insight into the upcoming number of births (Textbox 1, quote 1A), and the general need of care in the region. This meant that health care providers tried to provide as much care as possible by themselves, rather than collaborating with other health care providers in the region. Additionally, stakeholders stated that when it came to planning, they only used their own data and experiences, and that sharing of information was not common practice (Textbox 1, quotes 1B and 1C).

Textbox 1. **Quotes illustrating insights before the introduction of data from the Saltro and tool. Quotes are translations from original Dutch transcripts.**

**Quotes**

- 1A: We came out of a situation where there was less trust and more competition.
- 1B: We just did something, especially forecasts. We didn’t know a lot; we knew what we did ourselves.
- 1C: [We planned based] on our own numbers, not taking forecasts into account, because we didn’t predict anything

#### Insights After Introduction of the Tool

Stakeholders did not use the forecasts for day-to-day staff planning, as the interval of the forecasts was still considered too wide, and the uncertainty around the forecasts was considered too large, as most women give birth in a period of 5 weeks around the predicted date of birth (Textbox 2, quote 2A). Interestingly, the application stressed the increasing sense of urgency of high health care demand and the communication of this urgency to the management and higher-up levels. Additionally, this sense of urgency also resulted in the regional collaborations and a lower sense of competitiveness (Textbox 2, quote 2B).

Textbox 2. **Quotes illustrating further use of the tool and room for improvements and research with regards to the tool. Quotes are translations from original Dutch transcripts.**

**Quotes**

- 2A: The problem is that the forecast is done with a period of 5 weeks where women give birth, and then not even all of them give birth [during that time]
- 2B: We dealt with each other in a very different way, because we were each other's competitors and we kept our cards to our chests. That's completely gone.
- 2C: Saying: don’t take a holiday] is a difficult one, but we stopped schooling, training, several working groups, social activities, they were all canceled.
- 2D: On the one hand, you have the care demand with the forecasts, on the other hand, you have the care capacity, pretty high level, but it pretty well resembles the weekly reality. It made sure that we felt the urgency to have things together the first two weeks of December. So, canceling schooling, canceling all other stuff, make sure we are ready operationally.
- 2E: [We] have the sense of urgency to talk about this [timeliness], and it [the forecast] is a tool to get things rolling, which were otherwise ignored. Then everyone would have said: “We should look at it sometime.” Now it is: “We should look at it now”.

This sense of urgency also had implications for planning on the operational level. For example, during the summer and autumn of 2021, the number of births was relatively high, around 10% to 20% above average for that specific period. By using the forecasts, obstetricians were able to increase their number of regional escalation management meetings and subsequently de-escalate when the care demand decreased again in December. Additionally, the sense of urgency also resulted in the cancellation of schooling, working groups, and social activities. The tool helped gain insight into when education and holidays should be planned, moved, or canceled (Textbox 2, quotes 2C and 2D).

Finally, the tool created the insight and urgency to act on other markers for QoC. As the numbers of births increase over a time period, and the availability of resources decreases, the focus of care was not just on patient safety, but also on timeliness and availability of care (Textbox 2, quote 2E).

#### Other Means of Use

Another way in which the tool could be used and add value was in the communication of the available care toward clients and patients. The stakeholders indicated that, at present, prospective parents are selecting their preferred hospitals for childbirth. However, this preference cannot always be met, due to limited capacity of these hospitals (Textbox 3, quotes 3A and 3B)*,* especially since total capacity of hospitals is based on regional instead of local demand. The stakeholders considered the tool as a helpful tool to discuss expectations of pregnant women and to manage their expectations and preferences. Stakeholders also identified a current QoC issue in obstetrics, namely when pregnant women receiving extra obstetric care were not always able to deliver in that specific hospital, which was considered undesirable as stakeholders argued that these women should receive care from one single (specialized) hospital (Textbox 3, quote 3C).

Textbox 3. **Quotes illustrating further use of the tool and room for improvements and research with regards to the tool. Quotes are translations from original Dutch transcripts.**

**Quotes**

- 3A: The chance that a pregnant woman delivers in the hospital of their choice, let’s be positive, is about 60%.
- 3B: We can’t make that [choosing the hospital to deliver] happen anymore, we would want to, but we can’t make it happen. Using these data, we can tell them why, … and first-line obstetrics care (i.e. community midwives and postpartum caregivers) can profit from this, because they end up telling parents that they can’t deliver where they wanted to deliver, so that something that should be beautiful ends up being less beautiful.
- 3C: I think that we get to the point that we are going to talk to each other, that there are patients that we know and that should stay in our hospitals, because there is more at hand with these patients, and that there are patients that have an uncomplicated pregnancy, but develop something in the last weeks, that it doesn’t matter where these [patients] end up.
- 3D: We here are ‘believers’, but there are people who are not used to think in this way, and by showing these numbers over and over again, we show what we can do using these numbers.
- 3E: We don’t think together what we have available with regards to capacity, and we don’t share [this information] in the first line care what we have filled up with care demand.
- 3F: You know how many pregnancies there are, which is nice, but we don’t know where they will deliver, and how this relates to each midwifery practice.
- 3G: I think that postal code is the most important, the area of adherence, because that’s where we eventually, if we manage it, can tune the data for the obstetrics and maternity care.
- 3H: Based on historical data you can determine for some group when they will deliver, as long as you have them well monitored.
- 3I: Can you say anything about the percentages of [caesarian sections]? Because then you can also account for length of stay and impact on admission and staffing.
- 3J: We have the opportunity now, because COVID gives us this opportunity. If you look at the [National Coordination Centre for Patient Allocation], data is used differently. We have the opportunity to design something like that, maybe we should start with that regionally.

Another possible application was seen in the education of (health care) professionals in obstetrics, as data on health care demand are not widely shared. Stakeholders argued that, although they themselves were used to these kinds of tools, this was not the case for other health care professionals (Textbox 3, quote 3D). To create more familiarity with these kinds of tools, the stakeholders identified important hurdles. An example of a hurdle was the implementation of data sharing and central data collection. The stakeholders were not used to collaborating and sharing their data, and it was stressed this needed improvement (Textbox 3, quote 3E).

Stakeholders also commented on issues that were not addressed in our study, that is, features that would increase the usability and information density of the tool. Primarily, the stakeholders stated that more information could be gained with regard to specific patient groups: on localization of the patients (Textbox 3, quotes 3F and 3G) or subgroups to be included, e.g., cesarean sections ( Textbox 3, quotes 3H and 3I). Stakeholders identified that in order to implement some of these improvements for the tool, more data should be shared among stakeholders in the obstetrics field. For example, stakeholders proposed a centralized data collecting system, similar to the system as implemented in the Netherlands during the COVID-19 ( Textbox 3, quote 3J).

## Discussion

### Main Findings

In this study, we developed, validated, and implemented a tool to forecast and visualize trends of births in the greater Utrecht region, using routinely measured and collected calculated date of births by a primary care laboratory, to support management of the regional acute obstetric care, including staff planning. We developed a forecasting model and found that a 2- and 3-weekly binned forecasting model provided good predictive power when it comes to forecasting trends within birth numbers. Subsequently, we presented our findings to stakeholders in obstetrics and then implemented a tool to visualize our forecasts using a 2-weekly binned forecast. Although 3-weekly binned forecasts showed slightly higher predictive power, the choice for 2-weekly binned forecast was made, as this provided a more granular view of obstetric care demand. After 6 months, using an evaluative stakeholder meeting, we found that, although our tool was not used in day-to-day staff planning but rather on a (global) operational level, we were able to add value to the obstetrics field by adding a sense of urgency, resulting in improved regional collaborations and operational actions and improving QoC in obstetrics.

QoC is multifaceted. According to stakeholders, the use of our tool in daily practice enabled the focus on timeliness. Simultaneously, the tool supported collaborations in obstetric care, leading to more efficient and available obstetric care. A well-coordinated acute obstetric care chain is important, as impaired communication between health care providers is detrimental to patient safety and continuity of care [10].

We implemented the forecasting tool during the COVID-19 pandemic. This crisis sparked the demand for more insights into obstetric health care demand, especially since during the pandemic, births varied more than before the pandemic. Examples include a birth wave after lockdown and fewer preterm births during the first lockdown [11,12]. Similar to other literature, we found a decrease in birth numbers in 2020 and an increase in birth numbers in 2021. Additionally, the surge of COVID-19 required more flexible resource management, as resource requirements changed or were scarce [13]. Considering that our data are able to capture the true birth rate in the general population opens up additional angles for research. Further study could focus on the decline in birth rate as seen in Nordic countries and Germany after 2021 [14,15], by studying the trend in expected births in our data. The increase in care demand and need for flexible resource management due to the pandemic fueled the development and implementation of the tool, as regional resources had to be more efficiently allocated due to high demands. Stakeholders reported that our tool was helpful in achieving these aims, by offering concrete data that heightened the sense of urgency within the obstetric care.

The academic literature and practice alike highlight the challenges of forecasting acute care demand, as it is often driven by unforeseen events or factors that fall outside the scope of routine data collection [1,2]. Indeed, we observed in our data that it is very hard to forecast the weekly number of births, considering their weekly variance. However, widening the scope of the forecasting interval and retrieving information from these data, such as trends, are of added value to the obstetrics domain according to stakeholders in obstetric care.

Our research should be understood in the light of the following limitations. We have included only limited data, originating from municipalities for which data on at least 55% of pregnancies was available. The extent to which our study therefore suffers from selection bias remains unclear. As the obstetrics care in the Netherlands and the reporting of calculated date of births is standardized, we believe bias is limited. However, future studies could focus on combining data sources for the estimated dates of birth, in order to increase the data coverage, and to mitigate reliance on a single data source. Additionally, our dataset is relatively small. With larger numbers, the use of statistical models such as Autoregressive Integrated Moving Average, or machine learning models may be beneficial [16]. Considering we had data on the actual number of births for 3 years, the use of such models was currently infeasible. To the best of our knowledge, no other forecasting model for the number of births is currently used in the Netherlands.

As stakeholders have stated in the stakeholder meeting, there are several directions for further research. First of all, data should be shared between stakeholders to improve regional (acute) obstetric care. This could help further improve forecasts, as this can help identify subgroups of pregnancies, so that these groups and their health care demand can be further monitored. Additionally, data sharing could lead to further standardization of data collection, making data collection and research to improve obstetric care more feasible and impactful.

### Conclusion

Implementation of a forecasting tool for number of births based on available data across the health care system during the COVID-19 pandemic led to increased communication, awareness, and collaboration between obstetric health care providers, showing that combining several sources of data within the routine acute care can add value to the routine acute care process. Providing visualizations of forecasted births created a sense of urgency about the high care demand situation and the impact of this demand on quality of care. Our tool thereby facilitated improved collaborations and cohesion between health care providers in the Utrecht region and aided further initiatives that can increase efficient use of resources in response to high care demand beyond pandemic situations. Further research should aim at improving regional obstetric acute care by fostering data sharing in order to improve health care demand forecasts.

## Supplementary material

10.2196/68284 Multimedia Appendix 1 Topic list evaluative stakeholder meeting.

10.2196/68284 Multimedia Appendix 2 Predictions versus actual births in 2016 per week.

## Abbreviations:

QoC: quality of care
