# Supplementary material for: Development and Implementation of a Birth Forecasting Tool to Optimize Resources in Obstetrical Care During the COVID-19 Pandemic: Mixed-Methods Study
Source: JMIR Pediatr Parent. 2025 Aug 22;8:e68284. doi: 10.2196/68284 (PMC12373259; doi:10.2196/68284)
Supplement: Multimedia Appendix 1 [file pediatrics-v8-e68284-s001.docx]

***Topic List Evaluative Stakeholder Meetings***

| **Part 1: Situation before introduction of the numbers and tool** |
| --- |
| 1. *Did planning of regional capacity take place?*    1. *Was there any regional collaboration?* |
| 1. *Where did the information come from?* |
| 1. *Were high demand situations forecasted?* |
| **Part 2: After the introduction of the tool:** |
| 1. *How did the introduction of the birth numbers add value?* |
| 1. *Is there more insight into health care demand?* |
| 1. *How is the tool being used?*    1. *Is the tool being used (for planning)?*    2. *How is it used to acquire extra resources/re-distribute personel leave*    3. *Unforeseen changes introduced by the tool* |
| 1. *How are the forecasted birth numbers used in day to day work?* 2. *What is the added value of the forecasts?* |
| **Part 3: Regarding the tool itself** |
| 1. What do the numbers mean according to the stakeholders |
| 1. What does the average across earlier years add?    1. Is there any recollection regarding earlier years? |
| 1. What are improvements for the tool? |
