# Supplementary material for: Development and Implementation of a Birth Forecasting Tool to Optimize Resources in Obstetrical Care During the COVID-19 Pandemic: Mixed-Methods Study
Source: JMIR Pediatr Parent. 2025 Aug 22;8:e68284. doi: 10.2196/68284 (PMC12373259; doi:10.2196/68284)

Supplemental Figure 1: Predictions versus Actual Births in 2016 per week

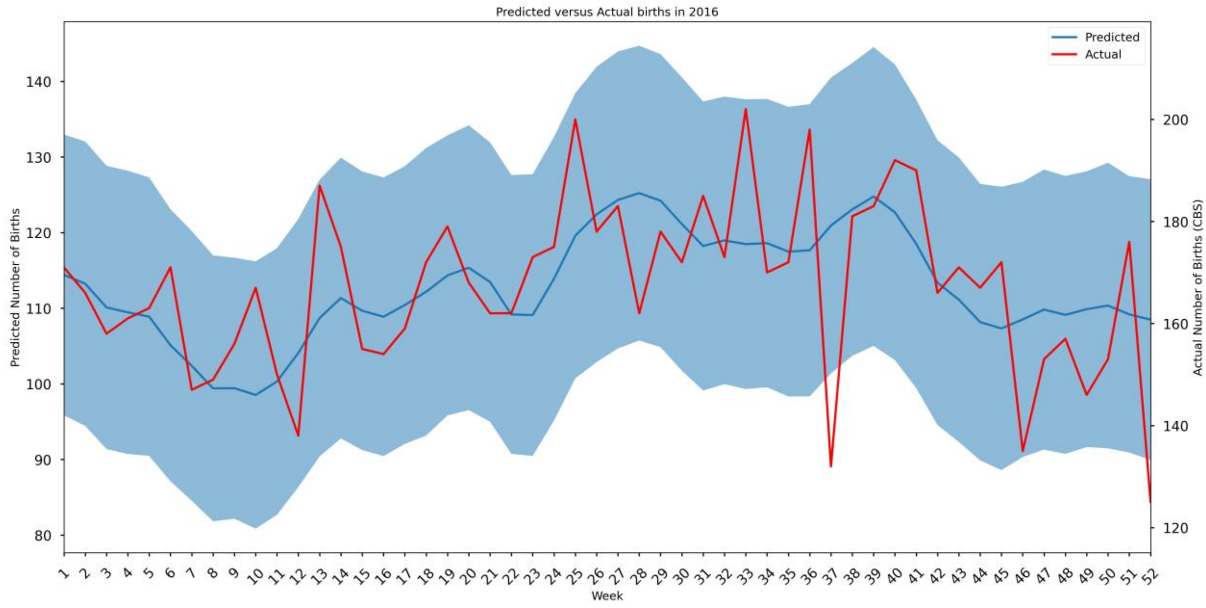

Supplemental Figure 2: Predictions versus Actual Births in 3 weekly bins

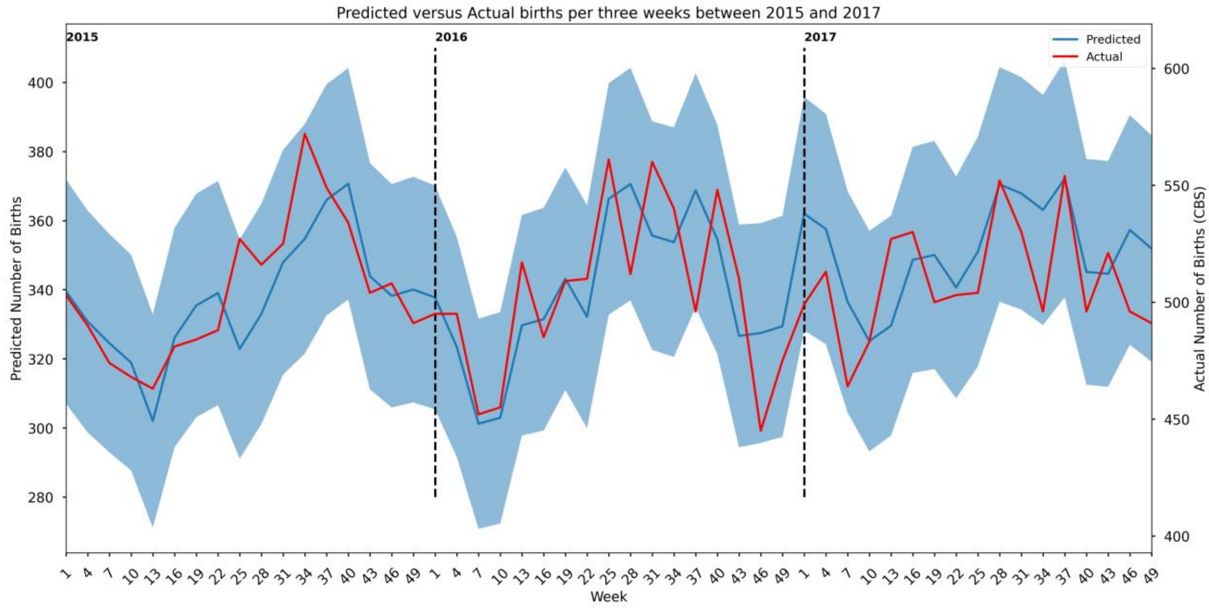

Supplement: Multimedia Appendix 2 [file pediatrics-v8-e68284-s002.pdf]
